# Coevolution of URAT1 and Uricase during Primate Evolution: Implications for Serum Urate Homeostasis and Gout

**DOI:** 10.1093/molbev/msw116

**Published:** 2016-06-26

**Authors:** Philip K. Tan, Jennifer E. Farrar, Eric A. Gaucher, Jeffrey N. Miner

**Affiliations:** ^1^Biology Department, Ardea Biosciences, Inc, San Diego, CA; ^2^School of Biology, Georgia Institute of Technology; ^3^General Genomics, Atlanta, GA

**Keywords:** URAT1, hyperuricemia, uric acid affinity, evolution.

## Abstract

Uric acid is the highly insoluble end-product of purine metabolism in humans. Serum levels exceeding the solubility threshold can trigger formation of urate crystals resulting in gouty arthritis. Uric acid is primarily excreted through the kidneys with 90% reabsorbed back into the bloodstream through the uric acid transporter URAT1. This reabsorption process is essential for the high serum uric acid levels found in humans. We discovered that URAT1 proteins from humans and baboons have higher affinity for uric acid compared with transporters from rats and mice. This difference in transport kinetics of URAT1 orthologs, along with inability of modern apes to oxidize uric acid due to loss of the uricase enzyme, prompted us to ask whether these events occurred concomitantly during primate evolution. Ancestral URAT1 sequences were computationally inferred and ancient transporters were resurrected and assayed, revealing that affinity for uric acid was increased during the evolution of primates. This molecular fine-tuning occurred between the origins of simians and their diversification into New- and Old-World monkey and ape lineages. Remarkably, it was driven in large-part by only a few amino acid replacements within the transporter. This alteration in primate URAT1 coincided with changes in uricase that greatly diminished the enzymatic activity and took place 27–77 Ma. These results suggest that the modifications to URAT1 transporters were potentially adaptive and that maintaining more constant, high levels of serum uric acid may have provided an advantage to our primate ancestors.

## Introduction

Serum uric acid (sUA) in humans is tightly regulated to a normal range of 210–420 µM (3.5–7 mg dl^−^^1^) (Feig et al. 2006). At higher concentrations, uric acid exceeds its solubility limit in biological fluids (Terkeltaub and Edwards 2013). Individuals with chronically elevated sUA levels (hyperuricemia) are prone to developing gout, a disease characterized by bouts of painful inflammatory arthritis due to crystallization of uric acid (monosodium urate) in the joints (Terkeltaub and Edwards 2013). In addition to its established role in gout, hyperuricemia has more recently been implicated as a risk factor for various metabolic, hemodynamic, and systemic disorders, including metabolic syndrome, obesity, diabetes, kidney disease, hypertension, stroke, and atherosclerosis (Zhu et al. 2012; Sattui et al. 2014).

Uric acid is the end product of purine catabolism in apes, including humans and certain monkeys, due to the inactivation of uricase, an enzyme that oxidizes insoluble uric acid into 5′-hydroxyisourate, which is ultimately converted into soluble allantoin. Applying ancestral sequence reconstruction to resurrect ancient uricases from early primate evolution suggests that the enzyme accumulated a series of amino acid replacements that rendered it inactive and this occurred before the gene itself accumulated substitutions to generate a pseudogene by way of premature stop-codons (Wu et al. 1994; Oda et al. 2002; Kratzer et al. 2014). This lack of activity is likely one reason why sUA levels in hominoids are generally 3–10 times higher than in other mammals. The gradual inactivation of uricase suggests a potentially adaptive process but this is difficult to confirm since the sequences have been evolving under a neutral model since pseudogenization. Regardless, the increased sUA may have provided benefits during the course of primate evolution.

The production of uric acid is balanced by its elimination, mainly in the urine. In humans, sUA levels are controlled by a complex system of renal transporters in the proximal tubule of the kidney (Kottgen et al. 2013). Uric acid is freely filtered at the glomerulus, then, over 90% is reabsorbed back into the bloodstream; and less than 10% of the filtered uric acid is eventually eliminated in urine (Keenan et al. 2012; Terkeltaub and Edwards 2013). Uric acid is reabsorbed primarily through the sequential activities of URAT1 and GLUT9a, transporters that move uric acid across the apical and basolateral membranes, respectively, in epithelial cells of the renal proximal tubule. Inactivating mutations in these transporters cause idiopathic renal hypouricemia, characterized by high renal excretion of uric acid and very low sUA levels (Enomoto et al. 2002; Anzai et al. 2008). These transporters are thus essential for the renal reabsorption of uric acid—a highly efficient process lending credence to the notion that uric acid is not merely a waste product of purine metabolism in humans.

The URAT1 transporters from human, rat, and mouse (h/r/mURAT1) have been previously characterized (Enomoto et al. 2002; Hosoyamada et al. 2004; Sato et al. 2011). However, no comparison across species has been conducted. We directly compared URAT1 orthologs in a quantitative affinity assay and show that hURAT1 and baboon URAT1 (bURAT1) both have a higher affinity for substrate than either m- or rURAT1. Further, the primate transporters have a lower overall transport capacity than the rodent orthologs. At higher than normal sUA levels, the reduced capacity of the primate transporters saturates the transport kinetics, so that the uric acid transport activity is not further increased. This saturation has the effect of increasing the renal elimination of uric acid when sUA levels are higher than normal, thereby quickly restoring sUA levels to normal. We then resurrected and analyzed ancestral primate and rodent URAT1 transporters and found that ancestral URAT1 in the primate lineage underwent a two-step functional change during evolution. Initially, ancient primate URAT1s accumulated amino acid replacements that greatly increased affinity for uric acid (by lowering *K*_m_), accumulating gain of function mutations. Subsequent amino acid replacements then converted the ancient proteins into low capacity transporters. The two-step evolutionary fine tuning of transporters, along with the concomitant decrease in ancient uricase activity (Kratzer et al. 2014), ultimately would allow a potential adaptive increase in the serum concentration of uric acid while at the same time preventing sUA from exceeding a level that risks crystallization of the molecule in the body (>6.8 mg dl^−^^2^) (Terkeltaub and Edwards 2013). One potential adaptive pressure for increased uric acid may be derived from the ability of uric acid to stimulate the conversion of fructose into fat storage, allowing our frugivorous ancestors to survive in a resource-constrained environment, particularly during the food-scarce Miocene epoch (Choi et al. 2014; Cicerchi et al. 2014; Kratzer et al. 2014).

## Results

**Fig. 1:**
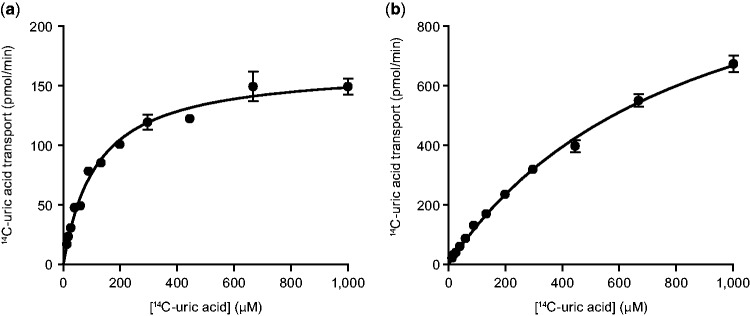
Human URAT1 is a high affinity/low capacity uric acid transporter. Uric acid transport activity for human (*a*) and rat (*b*) URAT1. Transfected HEK-293T cells were incubated for 1 min with different amounts of uric acid as described in Materials and Methods.

We measured the affinity (*K*_m_) and capacity (*T*_max_) of human, baboon, mouse, and rat URAT1 orthologs for uric acid in transiently transfected cells and provided quantitative evidence for species differences in affinity (fig. 1 and table 1). The affinity and capacity of hURAT1 and bURAT1 were similar, although bURAT1 had a slightly but significantly higher affinity (lower *K*_m_) than hURAT1 (table 1). The affinity of hURAT1 (*K*_m_ = 122 µM, fig. 1*a*) was 7-fold higher than rURAT1 (*K*_m_ = 857 µM, fig. 1*b*) and 5-fold higher than mURAT1 (*K*_m_ = 591 µM), while the transport capacity of hURAT1 was 9- and 7-fold lower than rURAT1 and mURAT1, respectively (table 1). This lower capacity indicates that hURAT1 will saturate more readily with high concentrations of uric acid. Normalization of the kinetic transport data from quantitative Western blotting (supplementary fig. S1, Supplementary Material online) demonstrates that hURAT1 is a high affinity and low capacity uric acid transporter compared with rURAT1.

**Table 1:** Summary of Uric Acid Transport Kinetics of URAT1 Constructs.

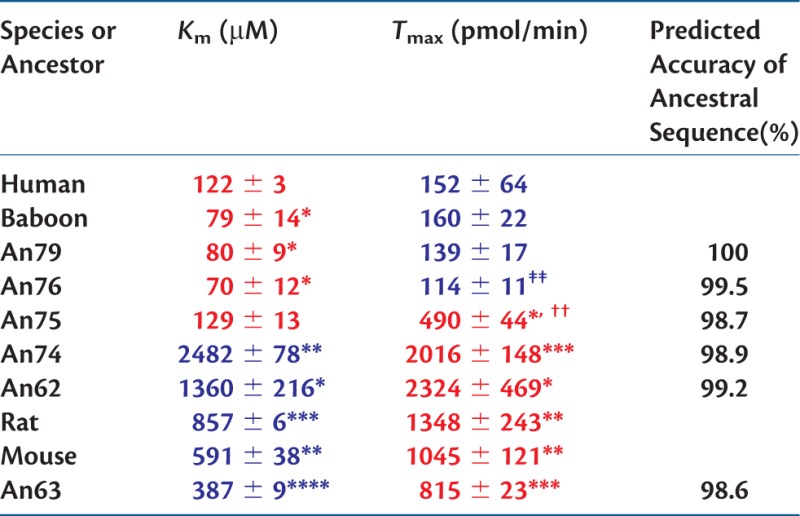

Notes.—Affinities in *K*_m_ and transport capacities in *T*_max_ were obtained from saturation transport curves, and are the mean of three experiments. Red represents high affinity (tight binding) and high capacity, whereas blue represents low affinity (weak binding) and low capacity.

Statistical analysis is based on the unpaired, two-tailed test.

*P < 0.05,

**P < 0.01,

***P < 0.001,

****P < 0.0001 compared with human.

^††^*P* < 0.001 compared with An74.

^‡‡^*P* < 0.01 compared with An75.

**Fig. 2:**
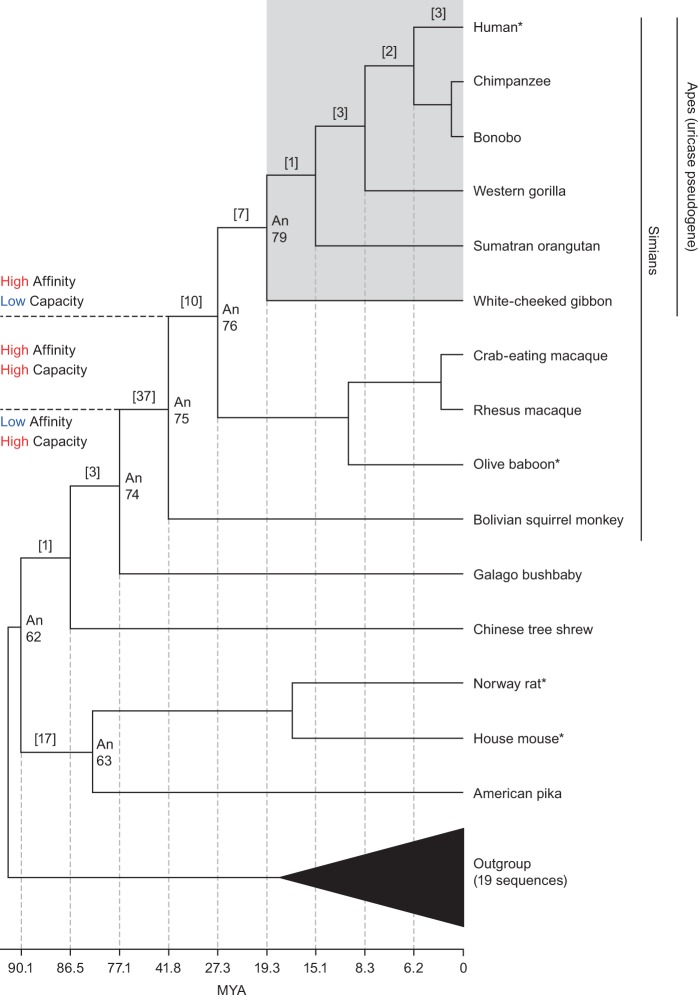
Phylogeny of the URAT1 transporter family and properties of modern and inferred ancestral transporters. Shown is a phylogenetic tree depicting the evolutionary relationships that focus on the Euarchontoglires portion URAT1 phylogeny. The nodes for which ancestral sequences were inferred and synthesized in the laboratory are labeled with the prefix of “An.” Modern transporters assayed in this study have an asterisk. The ape clade is boxed in gray and labeled to note the absence of functional *uricase* enzymes in this URAT1 phylogeny. The number of amino acid replacements between any two nodes is bracketed. Additional rodents/lagomorphs were used for evolutionary analyses but not shown in this simplified tree (supplementary fig. S3, Supplementary Material online). Divergence times for ancestral nodes are provided along the horizontal plane (Hedges et al. 2015). Color corresponds to values from table 1.

**Fig. 3:**
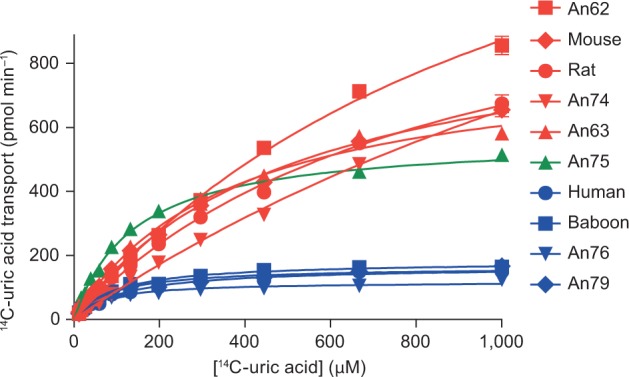
Uric acid transport kinetics of ancestral and extant URAT1 transporters. Assays were done as described in figure 1. High-affinity/low-capacity transporters, similar to hURAT1, are shown in blue. Low-affinity/high-capacity transporters, similar to r/mURAT1, are shown in red. The high-affinity/high-capacity transporter An75 is shown in green.

Next, we resurrected ancient URAT1 transporters from primate and rodent lineages that diverged between 90 and 20 Ma (Hedges et al. 2015) and analyzed uric acid transport kinetics for each (figs. 2 and 3 and table 1; see supplementary fig. S2, Supplementary Material online, for transporter protein sequences). All transporters were active and had a range of substrate affinities and transport capacities that aligned with their descendent proteins. An62, An63, and An74 displayed low affinity (*K*_m_) for uric acid but high capacity (*T*_max_) for substrate transport. This profile is similar to that of the rodent URAT1 transporters suggesting that up until the time of An74, URAT1 function remained unchanged (fig. 3, red curves, and table 1). A notable transition, however, occurred between An74 and An75, whereby An75 has a 19-fold “increase” in affinity and a 4-fold “decrease” in transport capacity for uric acid (fig. 3, green curve, and table 1).

**Fig. 4:**
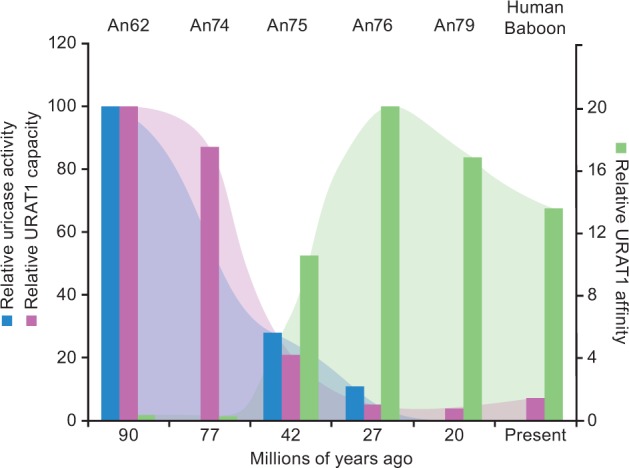
Gain of high uric acid affinity for URAT1 occurred at the same time in evolution as loss of uricase activity. Data for uricase activity were adapted from Kratzer et al. (2014) where the catalytic efficiency (*K*_cat_/*K*_m_) of uricase ancestor An19/22 (7.08 × 10^5^) from approximately 90 Ma was set to 100%. The catalytic efficiencies of other ancestral uricases are expressed as a percentage of the activity of An19/22. Uric acid affinity and transport capacity of URAT1 are from table 1 where, for An62, the *K*_m_ (1,360 µM) was set to 1.0 and the *T*_max_ (2,324 pmol/min) was set to 100. The affinity and transport capacity of the other URAT1 transporters were expressed as the ratio relative to An62.

Another transition between An75 and An76 completes the proposed two-step evolution of ancestral primate URAT1. An76 has an additional 4-fold reduced transport capacity compared with An75 (table 1). It is along this branch that mammalian URAT1 transporters definitively lose their high transport capacity property. The significant decrease in *T*_max_ causes these transporters to saturate earlier when presented with increasing uric acid concentrations. The observation that An76, An79, bURAT1, and hURAT1 transporters all display the same affinity/capacity profile (fig. 3, blue curves, and table 1) suggests that the conversion of low-affinity/high-capacity transporters to high-affinity/low-capacity transporters was completed by approximately 27 Ma. Figure 4 summarizes the relative affinity and transport capacity of URAT1 for uric acid in the Euarchontoglires clade (rodent and primate), alongside the relative catalytic efficiency of uricases. A 10-fold gain in affinity and 4-fold reduction in transport capacity for uric acid by URAT1 occurred by approximately 42 Ma, coinciding at a time when uricase lost nearly 70% of its catalytic efficiency (Kratzer et al. 2014). Another 4-fold reduction in transport capacity for uric acid by URAT1 occurred by approximately 27 Ma, when uricase lost nearly 90% of its catalytic efficiency. The transport kinetics of primate URAT1 then remained unchanged from approximately 27 Ma to the present, whereas uricase was completely inactivated by 20 Ma.

We next applied evolutionary algorithms capable of detecting changes in selection to better understand the phenotypic transitions between the ancestral transporters. Standard models of positive selection (both branch-based and site-based) failed to identify episodes of adaptive evolution (Yang et al. 2000; Yang and Bielawski 2000). However, the phylogenetic distribution of residues replaced along the branch connecting An74 to An75 highlighted sites potentially involved in functional divergence by displaying the constant-but-different pattern of amino acid distribution. This pattern arises when an otherwise conserved site is released of its selective pressure, residues are replaced, and then selection quickly reapplies pressure to be conserved (Gaucher et al. 2001 , 2002; Anisimova and Liberles 2007; Studer and Robinson-Rechavi 2010). Four sites of interest were identified as displaying complete conservation outside of the simian group while also being completely conserved within the simian group but having a different amino acid identity (sites 25, 27, 365, and 414).

These four sites replaced along the branch separating An74 from An75 were individually mutated in hURAT1 and/or rURAT1 to determine whether the changes affected binding affinity for uric acid. Table 2 shows that three of these sites, residues 365, 25, and 414, confer a tighter (higher) binding affinity for uric acid (lower *K*_m_) with the simian-specific residue than with the nonsimian residue. The importance of the residue at site 365 is shown by replacement of the nonsimian residue, tyrosine, with the simian residue, phenylalanine, in rURAT1. This rURAT1-Y365F transporter has an affinity for uric acid (*K*_m_ = 560 µM) that is 1.5-fold “higher” than rURAT1 wild-type (table 2). Conversely, the reverse replacement in hURAT1, resulted in the hURAT1-F365Y transporter having an affinity for uric acid (*K*_m_ = 280 µM) that is 2.3-fold “lower” than the wild-type hURAT1. These findings indicate that residue 365 is involved in substrate recognition, and that phenylalanine is important for the high-affinity uric acid binding by hURAT1. The simian to nonsimian replacements, M25V and L414V, also significantly reduced uric acid affinity, albeit with a smaller effect. None of these replacements significantly altered *T*_max_. Thus, these sites do not affect transport capacity within the background of an otherwise low-capacity transporter. hURAT1-S27P showed weak binding activity that could not be measured reliably.

**Table 2:** Summary Uric Acid Transport Kinetics of Human and Rat URAT1 Point Mutants.

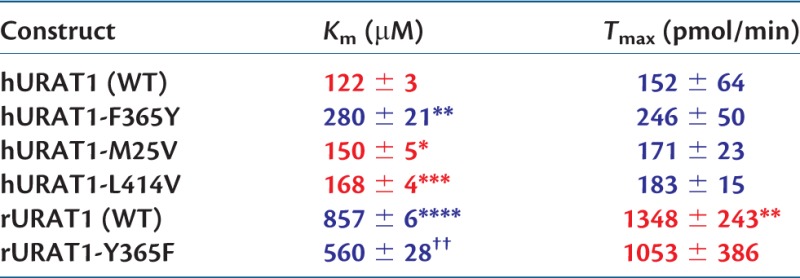

Notes.—See table 1 for details. Point mutants of hURAT1 were not compared with rURAT1 (WT), and rURAT1-Y365F was not compared with hURAT1 (WT). Red represents high affinity (tight binding) and high capacity, whereas blue represents low affinity (weak binding) and low capacity.

*P < 0.05,

**P < 0.01,

***P < 0.001,

****P < 0.0001, compared with hURAT1 (WT).

^††^*P* < 0.01 compared with rURAT1 (WT).

## Discussion

We have demonstrated using evolutionary inference and quantitative biochemical transport assays that URAT1 acquired both a higher affinity and a lower capacity for the transport of uric acid during primate evolution. The change in affinity is driven by the replacement of a few residues, most importantly the replacement of residue 365 from tyrosine to phenylalanine that enhanced the affinity of URAT1 for uric acid and appeared between approximately 77 and approximately 42 Ma, coinciding with the initial reduction in uricase activity that occurred in early primates. Uricase inactivation progressed stepwise in a number of independent events over the time frame of about 20 My (Kratzer et al. 2014). It is hypothesized that the gradual inactivation of uricase was necessary to better manage an increase in uric acid load (Kratzer et al. 2014), because mice engineered with homozygous deletions of the uricase gene die at a high rate after birth (Wu et al. 1994). We suggest that URAT1 coevolved with the inactivation of uricase and this occurred in a two-step manner. The first step of URAT1 divergence involved tighter-binding affinity and a decreased capacity for uric acid between 77and 42 Ma. During this same time, uricase was decreasing in catalytic efficiency by 3-fold. The second step of URAT1 adaptation involved a transition to further lower transport capacity for uric acid between 42 and 27 Ma. During this same time, uricase decreased in catalytic efficiency by another 3-fold. Most importantly, once the ancient simian URAT1 transporter evolved both high affinity and low capacity, only then could uricase accept the single-point mutation that completely abolished its catalytic efficiency (F222S), shortly after 27 My (Kratzer et al. 2014).

**Fig. 5:**
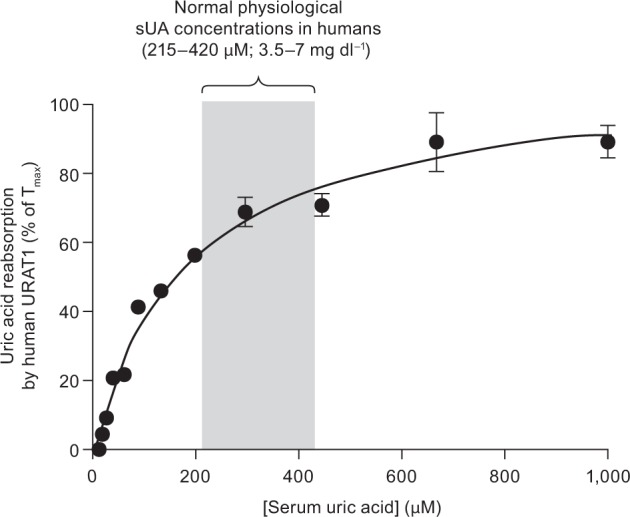
Human URAT1 evolution to regulate sUA levels. Results from figure 1*a* are shown, except the physiologic parameters of renal uric acid reabsorption and sUA concentrations are used to express the rate of uric acid transport relative to sUA concentration. At low uric acid levels (<215 µM), the high-affinity/low-capacity transporter is very active at uric acid reabsorption, whereas at high uric acid levels (>420 µM), the transporter is highly saturated and thus unable to transport most of the uric acid. This characteristic allows human URAT1 to more tightly control sUA levels. See Discussion for more details.

The consequence of the conversion of URAT1 to a high-affinity/low-capacity transporter is illustrated in figure 5. Here, the hURAT1 activity is placed in physiological context, in which the kinetics of uric acid transport are represented as the rate of renal uric acid reabsorption relative to the sUA concentration (which is the same as the concentration of glomerular-filtered uric acid). As a reference, the normal physiological sUA concentration range in humans (3.5–7 mg dl^−^^1^, or 215–420 µM) is shown (Feig et al. 2006). At low sUA levels, hURAT1 is highly active and efficiently reabsorbs uric acid, due to its high affinity. In contrast, at higher levels, hURAT1 is highly saturated as the transport rate reaches its *T*_max_, due to its low transport capacity. As a result of this low capacity, hURAT1 can quickly restore sUA levels after ingestion of a meal that produces uric acid, and the excess uric acid is excreted into the urine. Therefore, these changes in URAT1 for later primates allowed for greater uric acid retention and more precise control of sUA levels.

With an increase in affinity and a lowering of capacity, URAT1 became a key regulator for controlling sUA concentrations, permitting complete uricase inactivation during primate evolution. Humans with homozygous inactivating mutations in URAT1 have dramatically lower sUA levels (<1 mg dl^−^^1^) and high renal fractional excretion of uric acid (Enomoto et al. 2002) compared with humans with functional URAT1. This demonstrates that URAT1 is essential for maintaining the relatively high sUA levels in humans. Previous studies have suggested that uric acid is regulated by a large number of transporters in the human kidney (Kottgen et al. 2013). We anticipate that in addition to URAT1, evolutionary changes in the activity of these other uric acid transporters (e.g., GLUT9, BCRP, NPT1, NPT4, OAT4, MCT9) are likely to have occurred concomitantly.

The evolutionary pressure to increase uric acid levels may have driven selection for a number of stepwise changes in protein function over millions of years to accommodate higher sUA levels. These include limiting uric acid metabolism by decreasing uricase activity, while increasing the ability to reabsorb uric acid under low sUA loads. Further alterations in the saturation kinetics of ancestral transporters ensured that fluctuations in sUA would not achieve levels that could cause the uric acid to crystalize in the body.

We have demonstrated that during mammalian evolution, URAT1 has evolved from a low-affinity to a high-affinity uric acid transporter in a similar time frame as the loss of uricase activity––occurring with the rise of simians. These modifications to the urate handling system in the kidney started in the Paleocene/Eocene epochs, and were complete by the early Miocene (∼25 Ma) (Kratzer et al. 2014). During this time, there was progressive atmospheric cooling, and the rainforests receded to the equatorial zone (Johnson and Andrews 2010). The alterations in URAT1 and uricase resulted in an increase in, and stabilization of, sUA levels, and may have provided a survival advantage to our frugivorous primate ancestors in Europe and Asia by amplifying the effects of fructose to enhance fat stores as they followed the fruit back to Africa (Johnson and Andrews 2010). In modern times of excessive resources and fructose, these evolutionary changes may have inadvertently predisposed humans to gout, a metabolic disease caused by a disruption of sUA homeostasis. About 80–90% of gout patients have reduced renal fractional excretion of uric acid (Boss and Seegmiller 1979), which promotes chronic hyperuricemia. Recently, we have shown that gout patients have a uric acid reabsorption system that is less saturated compared with individuals with normal sUA levels (Liu et al. 2016) This difference can be explained by differences in the saturation kinetics of uric acid reabsorption. Understanding the reasons for these differences may ultimately lead to new therapies for gout.

## Materials and Methods

^14^C-uric acid (50–60 mCi mmol^−^^1^, 0.5 mCi/ml) was purchased from American Radiolabeled Chemicals, Inc.

### Generation of Ancestral URAT1 Sequences

Ancestral URAT1 sequences were inferred by analyzing modern transporter sequences within an evolutionary framework and followed our previous protocols (Gaucher et al. 2003 , 2008; Kratzer et al. 2014). Modern URAT1 sequences from 42 taxa were retrieved from public databases and aligned using MUSCLE. An evolutionary tree was inferred using MrBayes (Huelsenbeck et al. 2001). The following parameters were incorporated during the analysis: The generalized time reversible DNA substitution model, a proportion of the nucleotide sites were invariable while the remaining sites were drawn from a gamma distribution. To search tree-space, two independent Metropolis-coupled Markov chain Monte Carlo (MCMCMC) simulations, four chains each, were performed for 1,000,000 generations with parameter sampling every 100 generations. The first 100 samples were discarded during the burn-in phase of the MCMCMC analysis. The resulting gene tree recapitulated the species tree for these organisms.

Ancestral sequences and the nonsynonymous-to-synonymous ratio (d*N*/d*S*) were inferred using PAML v4.1 (Yang 2007). Ancestral sequences were inferred using both DNA-based and amino acid-based models. The DNA-based analysis incorporated the same parameters as above. The amino acid-based analysis incorporated a gamma distribution and the WAG (Whelan and Goldman) replacement matrix. These various analyses generated identical ancestral sequences. The inferred ancestral sequences displayed high posterior probabilities (supplementary fig. S3, Supplementary Material online) and were robust to alternative models used during the inference process (Anzai et al. 2008; Choi et al. 2014; Cicerchi et al. 2014). Codon-based analyses were performed to calculate d*N*/d*S* ratios using both branch-based and site-based models. These analyses incorporated a gamma distribution, codon frequencies F3×4, and a transition/transversion ratio. For the site-based method, M1/M2 and M7/M8 (beta vs. beta&*ω*) analyses were not significantly different according to a Likelihood Ratio Test. Further, Bayes Empirical Bayes did not identify any positively selected sites having a *P* > 95%.

### Constructs and Mutagenesis

hURAT1 (GenBank BC053348.1), rURAT1 (NCBI NM_001034943.1), and mURAT1 (NCBI NM_009203) genes were purchased from Origene Technologies, Inc. hURAT1 and rURAT1 were subcloned into pCMV6/neo using *Not*I, creating pCMV6/neo-hURAT1 and pCMV6/neo-rURAT1. Point mutants were produced by polymerase chain reaction or site-directed mutagenesis using the QuikChange Lightning Multi Site Directed Mutagenesis Kit (Agilent Technologies), using mutagenic primers shown in supplementary table S1, Supplementary Material online. All mutants were confirmed by DNA sequencing. Ancestral and baboon URAT1 sequences were synthesized by Blue Heron Biotech, LLC and cloned into pCMV6/neo using *Eco*RI and *Xba*I. All URAT1 protein sequences are show in supplementary figure S2, Supplementary Material online.

### Cell Culture and Transfection

HEK-293T cells were maintained in Dulbecco’s Modified Eagle’s Medium/high glucose/l-glutamine/2-[4-(2-hydroxyethyl)piperazin-1-yl]ethanesulfonic acid (HEPES) supplemented with 1 mM sodium pyruvate (Life Technologies) and 10% fetal bovine serum (GE Healthcare Life Sciences), at 37 °C in 5% CO_2_. Plasmids were reverse transfected into HEK-293T cells. Ten micrograms of DNA was mixed with 30 µl of DreamFect Gold (Boca Scientific) in 2 ml OptiMem (Life Technologies). After 20 min, 18 ml of media containing 2E7 cells in suspension was added and mixed, and 200 µl per well was plated onto white clear-bottomed poly-d-lysine coated 96-well plates (BD Biosciences). The cells were assayed the next day.

### Transporter Activity Assays

Activity assays were performed using different amounts of ^14^C-uric acid in an assay buffer consisting of 25 mM 2-(N-morpholino)ethanesulfonic acid (MES) (adjusted to pH 5.5 with sodium hydroxide), 125 mM sodium gluconate, 4.8 mM potassium gluconate, 1.2 mM monobasic potassium phosphate, 1.2 mM magnesium sulfate, 1.3 mM calcium gluconate, and 5.6 mM glucose. URAT1 expressing cells were rinsed in 25 mM MES pH 5.5/125 mM sodium gluconate (wash buffer) and then incubated with 100 µM ^14^C-uric acid solution for 1 min. The transport rate of uric acid was linear for this incubation time (data not shown). Transport was then stopped by washing the cells three times in wash buffer. The cells were then solubilized in Ultima Gold (Perkin Elmer) prior to liquid scintillation counting. Each treatment was measured in triplicate. Nonspecific transport was measured in each plate in cells transfected with pCMV6/neo (Origene), and this was subtracted from the total transport to obtain the URAT1-specific transport. Nonlinear regression analysis using GraphPad Prism 5 software was used to determine the kinetics of uric acid transport from one site—specific binding equations. Statistical significance of uric acid affinities (*K*_m_ values) and transport capacity (*T*_max_ values) was assessed using the Student’s unpaired *t*-test. When variances were significantly different, the less stringent Welch’s correction was used.

### Western Blotting

For each hemagglutinin peptide (HA)-tagged URAT1 construct, 5E6 reverse transfected cells were plated into a T75 flask. On the following day, the cells were harvested using PBS containing 5 mM ethylenediaminetetraacetic acid (EDTA), pelleted, and resuspended in 1 ml of ice-cold membrane buffer (25 mM HEPES pH 7.3 [USB Biologicals, Inc], 125 mM sodium gluconate, and complete EDTA-free protease inhibitor cocktail [Sigma]). Cells were lysed with 80 strokes in a 2-ml dounce homogenizer. The lysates were transferred to a 1.5-ml Eppendorf tube and centrifuged at 250 g for 5 min at 2 °C. The supernatants were transferred to another Eppendorf tube and centrifuged again at 18,000 g for 20 min at 2 °C. The pellets were resuspended in solubilization buffer (Licor Biosciences protein loading buffer at 1X, containing 2.5% β-mercaptoethanol). Solubilized membranes were incubated at 70 °C for 10 min prior to electrophoresis. Electrophoresis and transfer of the solubilized membranes to nitrocellulose were done with Life Technologies’ NuPAGE system according to the manufacturer’s instructions, using 4–12% Bis-Tris gels and MES sodium dodecyl sulfate (SDS) running buffer. Western blotting was performed with blocking buffer and secondary antibodies conjugated to infrared dyes (Licor Biosciences) according to the manufacturer’s instructions, using mouse monoclonal antibody HA.11 (Covance) to detect HA-tagged URAT1 constructs, and rabbit polyclonal antibody ab8227 (Abcam) to detect β-actin. Proteins were visualized and quantified using an Odyssey Sa imaging system (Licor Biosciences). The signal for β-actin was used to normalize the protein levels for the calculation of URAT1 activities in figure 1a.

## Supplementary Material

Supplementary figures S1–S3 and table S1 are available at *Molecular Biology and Evolution* online (http://www.mbe.oxfordjournals.org/).

Supplementary Data
