# Supplementary material for: Coevolution of URAT1 and Uricase during Primate Evolution: Implications for Serum Urate Homeostasis and Gout
Source: Mol Biol Evol. 2016 Jun 26;33(9):2193–200. doi: 10.1093/molbev/msw116 (PMC4989112; doi:10.1093/molbev/msw116)
Supplement: Supplementary Data [file supp_33_9_2193__index.html]

Supplementary Data 

# Coevolution of URAT1 and Uricase during Primate Evolution: Implications for Serum Urate Homeostasis and Gout

## Supplementary Data

files

- Supplementary Data - pdf file
