# Supplementary material for: Coevolution of URAT1 and Uricase during Primate Evolution: Implications for Serum Urate Homeostasis and Gout
Source: Mol Biol Evol. 2016 Jun 26;33(9):2193–200. doi: 10.1093/molbev/msw116 (PMC4989112; doi:10.1093/molbev/msw116)
Supplement: Supplementary Data [file supp_msw116_TAN_MBE_Supplemental_Material.pdf]

Coevolution of URAT1 and uricase during primate evolution: implications for serum urate homeostasis and gout

Supplemental Figures and Tables

Supplemental Fig. S1. Expression of human and rat URAT1.

Western blot of hemagglutinin (HA) epitope-tagged human and rat URAT1. (**a**) The HA epitope was inserted at the same position both constructs. Plasma membrane enriched fractions from transfected cells were blotted and probed with antibodies to HA and actin. Quantitation of the signal showed that rat URAT1 wild type is expressed at 2.62-fold higher levels than human URAT1. (**b**) Kinetic transport data of human URAT1 (red) and rat URAT1 (green), normalized to the protein expression levels obtained from the quantitative Western blot in (**a**). Human URAT1 is a high affinity/low capacity transporter relative to rat URAT1. Kinetic transport data (**b**) of human URAT1 (red) and rat URAT1 (green), normalized to the protein expression levels obtained from the quantitative Western blot in (a). Human URAT1 is a high affinity/low capacity transporter relative to rat URAT1.

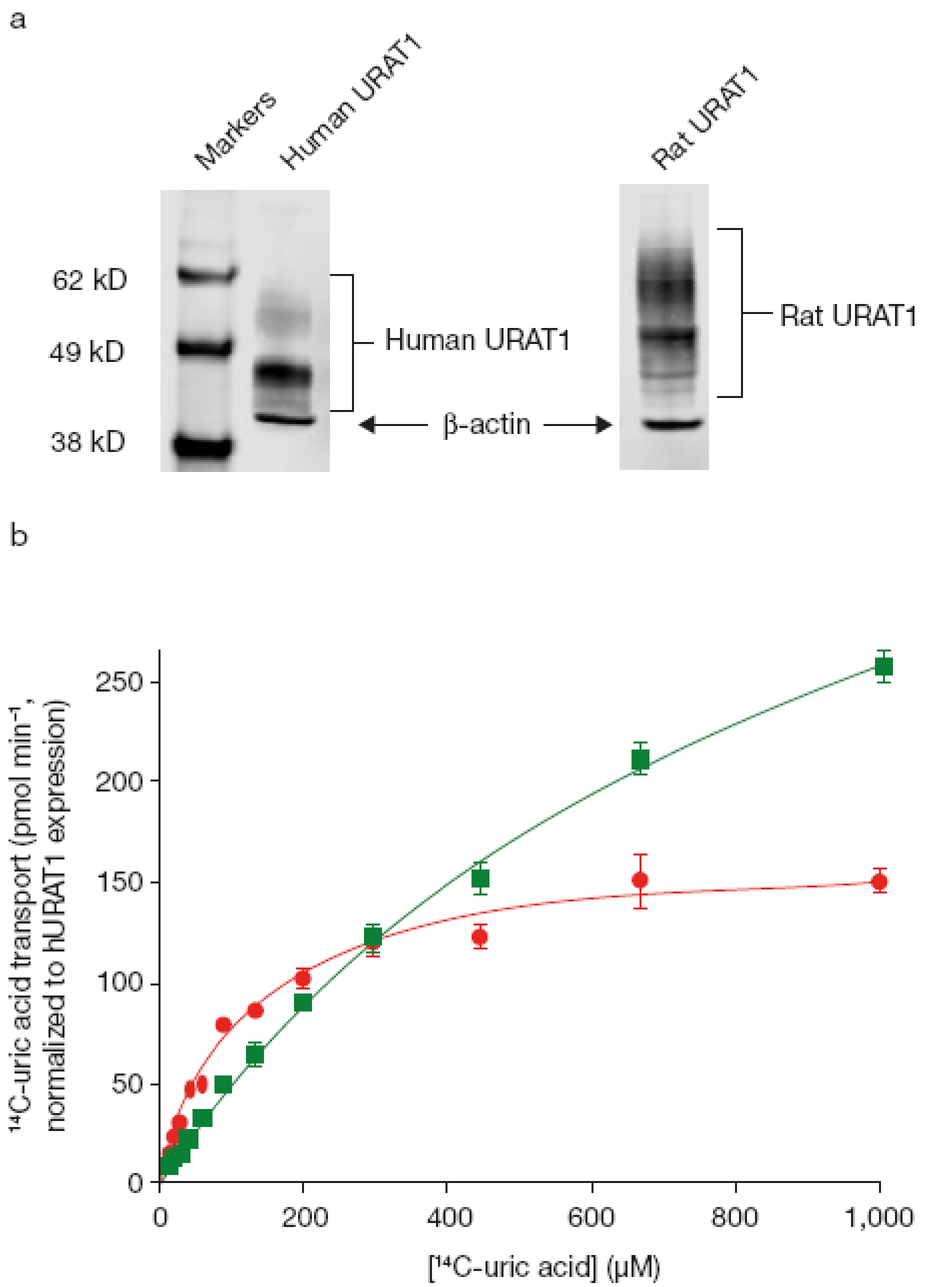
a
Markers
Human URAT1
Rat URAT1
62 kD
49 kD
38 kD
Human URAT1
β-actin
Rat URAT1
b
¹⁴C-uric acid transport (pmol min⁻¹, normalized to hURAT1 expression)
250
200
150
100
50
0
0
200
400
600
800
1,000
[¹⁴C-uric acid] (μM)

Supplemental Fig. S2. Protein sequences of URAT1 transporters used in this study.

Human URAT1
MAFSELLDLVGGLGRFQVLQTMALMVSIMWLCTQSMLENFSAAVPSHRCWAPLLDNSTAQASILGSLSPE
ALLAISIPPGPNQRPHQCRRFRQPQWQLLDPNATATSWSEADTEPCVDGWVYDRSIFTSTIVAKWNLVCD
SHALKPMAQSIYLAGILVGAAACGPASDRFGRRLVLTWSYLQMAVMGTAAAFAPAFPVYCLFRFLLAFAV
AGVMMNTGTLLMEWTAARARPLVMTLNSLGFSFGHGLTAAVAYGVRDWTLLQLVVSVPFFLCFLYSWWLA
ESARWLLTTGRLDWGLQELWRVAAINGKGAVQDTLTPEVLLSAMREELSMGQPPASLGTLLRMPGLRFRT
CISTLCWFAFGFTFFGLALDLQALGSNIFLLQMFIGVVDIPAKMGALLLLSHLGRRPTLAASLLLAGLCI
LANTLVPHEMGALRSALAVLGLGGVGAAFTCITIYSSELFPTVLRMTAVGLGQMAARGGAILGPLVRLLG
VHGPWLPLLVYGTVPVLSGLAALLLPETQSLPLPDTIQDVQNQAVKKATHGTLGNSVLKSTQF

Rat (Rattus norvegicus) URAT1
MAFPELLDRVGGRGRFQLLQAVALVTPILWVTTQNMLENFSAAVPHHRCWVPLLDNSTSQASIPGDFGRD
VLLAVSIPPGPDQRPHQCLRFRQPQWQLIESNTTATNWSDADTEPCEDGWVYDHSTFRSTIVTTWDLVCD
SQALRPMAQSIFLAGILVGAAVCGHASDRFGRRRVLTWSYLLVSVSGTIAALMPTFPLYCLFRFLVASAV
AGVMMNTASLLMEWTSAQAGPLMMTLNALGFSFGQVLTGSVAYGVRSWRMLQLAVSAPFFLFFVYSWWLP
ESARWLITVGRLDQSLRELQRVAAVNRRKAEADTLTVEVLRSAMQEEPNGNQAGARLGTLLHTPGLRLRT
FISMLCWFAFGFTFYGLALDLQALGSNIFLLQALIGIVDLPVKMGSLLLLSRLGRRLCQASSLVLPGLCI
LANILVPREMGILRSSLAVLGLGSLGAAFTCVTIFSSELFPTVIRMTAVGLGQVAARGGAMLGPLVRLLG
VYGSWLPLLVYGVVPVLSGLAALLLPETKNLPLPDTIQDIQKQSVKKVTHDIAGGSVLKSARL

Mouse (Mus musculus)URAT1
MAFPELLDRVGGLGRFQLFQTVALVTPILWVTTQNMLENFSAAVPHHRCWVPLLDNSTSQASIPGDLGPD
VLLAVSIPPGPDQQPHQCLRFRQPQWQLTESNATATNWSDAATEPCEDGWVYDHSTFRSTIVTTWDLVCN
SQALRPMAQSIFLAGILVGAAVCGHASDRFGRRRVLTWSYLLVSVSGTAAAFMPTFPLYCLFRFLLASAV
AGVMMNTASLLMEWTSAQGSPLVMTLNALGFSFGQVLTGSVAYGVRSWRMLQLAVSAPFFLFFVYSWWLP
ESARWLITVGKLDQGLQELQRVAAVNRRKAEGDTLTMEVLRSAMEEEPSRDKAGASLGTLLHTPGLRHRT
IISMLCWFAFGFTFYGLALDLQALGSNIFLLQALIGIVDFPVKTGSLLLISRLGRRLCQVSFLVLPGLCI
LSNILVPHGMGVLRSALAVLGLGCLGGAFTCITIFSSELFPTVIRMTAVGLCQVAARGGAMLGPLVRLLG
VYGSWMPLLVYGVVPVLSGLAALLLPETKNLPLPDTIQDIQKQSVKKVTHDTPDGSILMSTRL

Baboon (Papio anubis)URAT1
MAFSELLDLVGGLGRFQVLQTVALMVSIMWLSTQSMLENFSAAVPSHRCWVPLLDNSTAQAGVPGGLTPE
ALLAVSIPPGPNQGPHQCRRFRQPQWQILDPNATATSWSEADTEPCVDGWVYDRSIFTSTIVAKWNLVCD
SHALKPMAQSIYLAGILVGAAACGPASDRFGRRLVLTWSYLQMAVMGTAAAFAPTFPVYCLFRFLLAFAV
AGVMMNTGTLLMEWTAAQARPLVMTLNSLGFSFGHGLTAAVAYGVRDWTLLQLVVSVPFFLCFLYSWWLA
ESARWLLTTGRLDWGLRELWRVAAINGKGTVRDTLTPEVLLSAMREELSMDQAPASLGTLLRTPGLRFRT
CISMLCWFAFGFTFFGLALDLQALGSNIFLLQMFIGVVDIPAKMGALLLLSRLGRRPTQAASLLLAGLCI
LANTLVPHELGAVRSALAVLGLGGVGAAYTCITIYSSELFPTVLRMTAVGLGQMAARAGAILGPLVRLLG
VHGPWLPLLVYGTVPVLSGLAALLLPETQSLPLPDTIQDVQNQVVKKTTHGTLGNSVLKSTQF

An62
MAFSELLDQVGGLGRFQVLQTVALVVPIMWLTTHNMLENFSAAVPSHRCWVPLLDNSTAQASVPGALDPE
ALLAVSIPPGPNQGPHQCRRFRQPQWQLLDPNATATNWSEAATEPCVDGWVYDHSTFTSTIVTKWDLVCD

SQALKPMAQSIYLAGILVGAAVCGHASDRFGRRLVLTWSYLQMAVSGTAAAFAPTFPVYCLFRFLVAFAV
AGVMMNTGTLLMEWTSAQARPLAMTLNSLGFSFGQVLMAAVAYGVRDWALLQLAVSAPFFLCFVYSWWLA
ESARWLLITGRLERGLQELQRVAAINGKRAVGDTLTIEVLLSAMQEELSVGQAPASLGTLLRTPGLRLRT
CVSTLCWFAFGFTFYGLALDLQALGSNIFLLQVLIGVVDIPAKMGTLLLLSRLGRRPTQAASLVLAGLCI
LANTLVPHEMGALRSALAVLGLGGVGAAFTCITIYSGELFPTVLRMTAVGLGQMAARGGAILGPLVRLLG
VHGPSLPLLVYGTVPVLSGLAALLLPETQSLPLPDTIQDVQNQAVKKATHSTQGHSVLKSTRL

An63

MAFSELLDQVGGLGRFQVLQTVALVVPIMWLTTHNMLENFSAAVPSHRCWVPLLDNSTAQASVPGALDPE
ALLAVSIPLGPNQQPHQCRRFRQPQWQLLDPNATATNWSEAATEPCVDGWVYDHSTFTSTIVTKWDLVCD
SQALKPMAQSIYLAGILVGAAVCGHASDRFGRRLVLTWSYLQMAVSGTAAAFAPTFPLYCLFRFLVAFAV
AGVMMNTGTLLMEWTSAQARPLMMTLNSLGFSFGQVLMAAVAYGVRDWALLQLAVSAPFFLCFVYSWWLP
ESARWLLITGKLERGLQELQRVAAINGKRAAGDTLTMEVLLSAMQEELSGGQAPASLGTLLRTPGLRLRT
CVSMLCWFAFGFTFYGLALDLQALGSNIFLLQVLIGVVDLPAKMGTLLLLSRLGRRPSQAASLVLPGLCI
LANTLVPHEMGALRSALAVLGLGSVGAAFTCITIYSGELFPTVLRMTAVGLGQMAARGGAILGPLVRLLG
VHGPSLPLLVYGTVPVLSGLAALLLPETQNLPLPDTIQDVQNQAVKKATHSTPGHSVLKSTRL

An74

MAFSELLDQVGGLGRFQVLQTVALVVPIMWLTTHNMLENFSAAVPSHRCWVPLLDNSTAQASVPGALDPE
ALLAVSIPPGPNQGPHQCRRFRQPQWQLLDPNATATNWSEAATEPCVDGWVYDHSTFTSTIVTKWDLVCD
SQALKPMAQSIYLAGILVGAAVCGPASDRFGRRLVLTWSYLQMAVSGTAAAFAPTFPVYCLFRFLLAFAV
AGVMMNTGTLLMEWTSAQARPLVMTLNSLGFSFGQVLMAAVAYGVRDWALLQLAVSAPFFLCFVYSWWLA
ESARWLLTTGRLERGLQELQRVAAINGKRAVGDTLTIEVLLSAMQEELSVGQAPASLGTLLRTPGLRLRT
CVSTLCWFAFGFTFYGLALDLQALGSNIFLLQVLIGVVDIPAKMGTLLLLSRLGRRPTQAASLVLAGLCI
LANTLVPHEMGALRSALAVLGLGGVGAAFTCITIYSGELFPTVLRMTAVGLGQMAARGGAILGPLVRLLG
VHGPSLPLLVYGTVPVLSGLAALLLPETQSLPLPDTIQDVQNQAVKKATHSTQGHSVLKSTRL

An75

MAFSELLDLVGGLGRFQVLQTVALMVSIMWLCTQNMLENFSAAVPSHRCWVPLLDNSTAQAGVPGGLSPE
ALLAVSIPPGPNQRPHQCRRFRQPQWQLLDPNATATSWSEADTEPCVDGWVYDRSTFTSTIVTKWDLVCD
SHALKPMAQSIYLAGILVGAAACGPASDRFGRRLVLTWSYLQMAVMGTAAAFAPTFPVYCLFRFLLAFAV
AGVMMNTGTLLMEWTAAQARPLVMTLNSLGFSFGHGLTAAVAYGVRDWTLLQLAVSVPFFLCFLYSWWLA
ESARWLLTTGRLDRGLQELWRVAAINGKGAVQDTLTPEVLLSAMQEELSMGQAPASLGTLLRTPGLRLRT
CISTLCWFAFGFTFFGLALDLQALGSNIFLLQMLIGVVDIPAKMGTLLLLSRLGRRPTQAASLLLAGLCI
LANTLVPHEMGALRSALAVLGLGGVGAAFTCITIYSGELFPTVLRMTAVGLGQMAARGGAILGPLVRLLG
VHGPWLPLLVYGTVPVLSGLAALLLPETQSLPLPDTIQDVQNQAVKKATHGTLGNSVLKSTRF

An76

MAFSELLDLVGGLGRFQVLQTVALMVSIMWLCTQSMLENFSAAVPSHRCWVPLLDNSTAQAGVPGGLSPE
ALLAVSIPPGPNQRPHQCRRFRQPQWQLLDPNATATSWSEADTEPCVDGWVYDRSIFTSTIVAKWNLVCD
SHALKPMAQSIYLAGILVGAAACGPASDRFGRRLVLTWSYLQMAVMGTAAAFAPTFPVYCLFRFLLAFAV
AGVMMNTGTLLMEWTAAQARPLVMTLNSLGFSFGHGLTAAVAYGVRDWTLLQLVVSVPFFLCFLYSWWLA
ESARWLLTTGRLDRGLQELWRVAAINGKGAVQDTLTPEVLLSAMREELSMGQAPASLGTLLRTPGLRFRT
CISTLCWFAFGFTFFGLALDLQALGSNIFLLQMFIGVVDIPAKMGALLLLSRLGRRPTQAASLLLAGLCI
LANTLVPHEMGALRSALAVLGLGGVGAAFTCITIYSSELFPTVLRMTAVGLGQMAARGGAILGPLVRLLG
VHGPWLPLLVYGTVPVLSGLAALLLPETQSLPLPDTIQDVQNQAVKKATHGTLGNSVLKSTQF

An79

MAFSELLDLVGGLGRFQVLQTVALMVSIMWLCTQSMLENFSAAVPSHRCWAPLLDNSTAQAGVLGGLSPE
ALLAISIPPGPNQRPHQCRRFRQPQWQLLDPNATATSWSEADTEPCVDGWVYDRSIFTSTIVAKWNLVCD
SHALKPMAQSIYLAGILVGAAACGPASDRFGRRLVLTWSYLQMAVMGTAAAFAPAFPVYCLFRFLLAFAV
AGVMMNTGTLLMEWTAARARPLVMTLNSLGFSFGHGLTAAVAYGVRDWTLLQLVVSVPFFLCFLYSWWLA
ESARWLLTTGRLDRGLQELWRVAAINGKGAVQDTLTPEVLLSAMREELSMGQAPASLGTLLRMPGLRFRT
CISTLCWFAFGFTFFGLALDLQALGSNIFLLQMFIGVVDIPAKMGALLLLSRLGRRPTLAASLLLAGLCI
LANTLVPHEMGALRSALAVLGLGGVGAAFTCITIYSSELFPTVLRMTAVGLGQMAARGGAILGPLVRLLG
VHGPWLPLLVYGTVPVLSGLAALLLPETQSLPLPDTIQDVQNQAVKKATHGTLGNSVLKSTQF

Supplemental Fig. S3. Phylogram of URAT1s based on amino acid analysis. Internal nodes are labeled with the posterior probability for each ancestral URAT1 sequence inferred. The scale bar represents 0.04 amino acid replacements per unit evolutionary time (except for the branches leading to Myotis and Monodelphia which have been shortened for display purposes). GenBank Identifier (GI) numbers are provided after each species name.

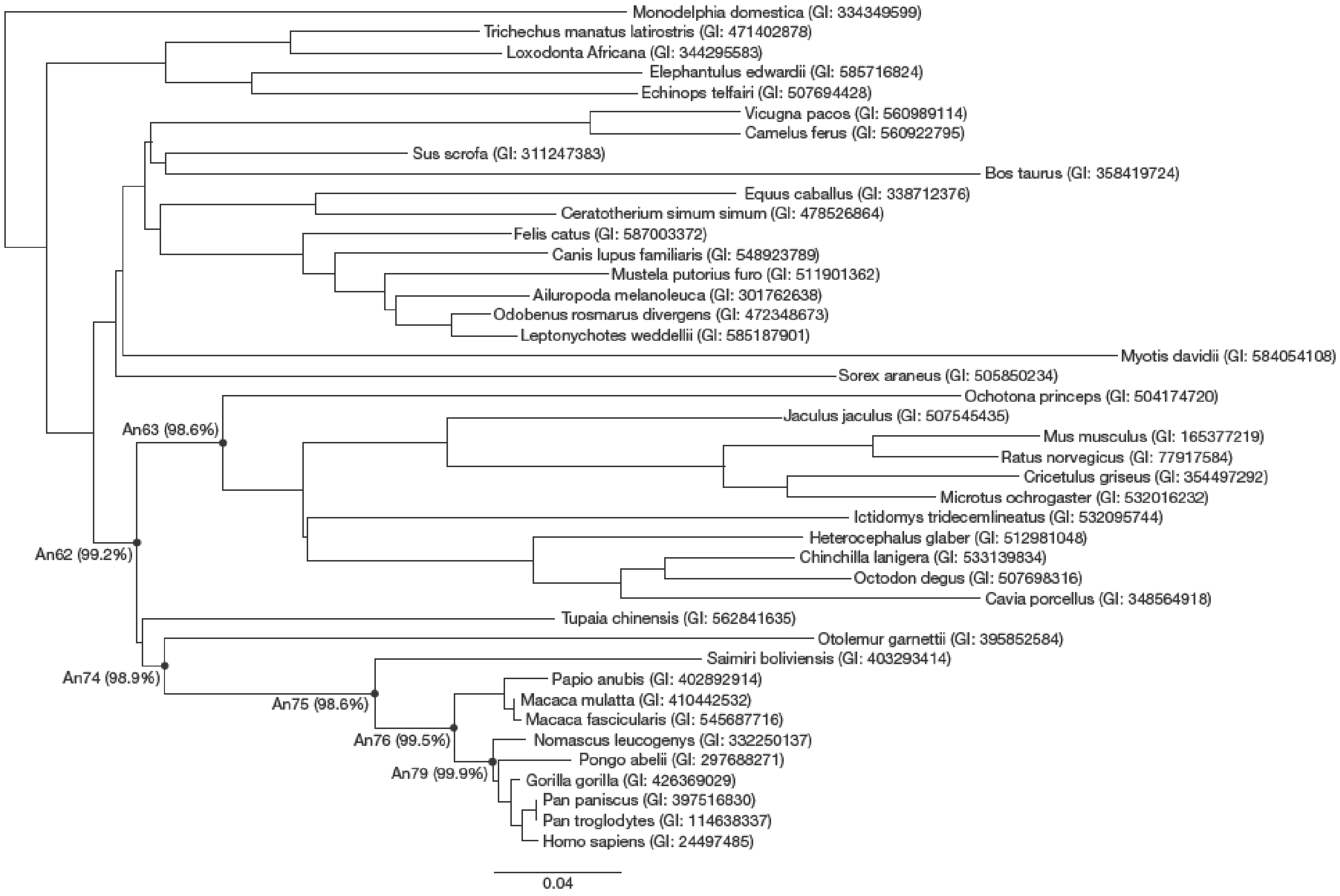

Supplemental Table S1. Mutagenic primers used in this study.

Name	Sequence	Purpose
h-F365Y	CCTTTGGCTTCACCTTCT**A**C GG**G**CT**A**GCCCTGGACCTGC AGGCC	Production of hURAT1-F365Y; marked with *Nhe*I site
h-M25V	GTTCCAGGTTCTCCAGAC**C**A TGGCTCTG**G**TGGTCTCCATC ATGTGG	Production of hURAT1-M25V, marked with site *Nco*I and *Xcm*I sites
h-S27P	CGATGGCTCTGATGGT**AC**CC ATCATGTGGCTGTG	Production of hURAT1-S27P, marked with *Kpn*I site
h-L414V	CAGAGCCCCGCCAGCA**C**CA **A**G**CT**TGCGGCCAGCGTGGG	Production of hURAT1-L414V, marked with *Hind*III site
r-Y365F	CCTTTGGCTTCACCTTCT**T**T GG**G**CT**A**GCCCTTGACCTGC AAGC	Production of rURAT1-Y365F; marked with *Nhe*I site
h-HA	AAGGCAACACATGGCTATCC CTACGACGTCCCTGATTATG CTACGCTGGGGAACTCTGTC	Production of hURAT1-HA (insertion of HA sequence after amino acid 541)
r-HA	GAAAGTGACACATGACTATC CCTACGACGTCCCTGATTAT GCTATAGCAGGCGGCTCCG	Production of rURAT1-HA (insertion of HA sequence after amino acid 541).

Residues in bold are modified from wild type sequences. Primers for insertion of the HA epitope tag all carry the sequence TATCCCTACGACGTCCCTGATTATGCT, encoding the peptide sequence YPYDVPDYA.
